# Are Sport-Specific Profiles of Tendon Stiffness and Cross-Sectional Area Determined by Structural or Functional Integrity?

**DOI:** 10.1371/journal.pone.0158441

**Published:** 2016-06-30

**Authors:** Hans-Peter Wiesinger, Florian Rieder, Alexander Kösters, Erich Müller, Olivier R. Seynnes

**Affiliations:** 1 Department of Sport Science and Kinesiology, University of Salzburg, Salzburg, Austria; 2 Department of Physical Performance, Norwegian School of Sport Sciences, Oslo, Norway; Université de Technologie de Compiègne, FRANCE

## Abstract

The present study aimed to determine whether distinct sets of tendon properties are seen in athletes engaged in sports with contrasting requirements for tendon function and structural integrity. Patellar and Achilles tendon morphology and force-deformation relation were measured by combining ultrasonography, electromyography and dynamometry in elite ski jumpers, distance runners, water polo players and sedentary individuals. Tendon cross-sectional area normalized to body mass^2/3^ was smaller in water polo players than in other athletes (patellar and Achilles tendon; -28 to -24%) or controls (patellar tendon only; -9%). In contrast, the normalized cross-sectional area was larger in runners (patellar tendon only; +26%) and ski jumpers (patellar and Achilles tendon; +21% and +13%, respectively) than in controls. Tendon stiffness normalized to body mass^2/3^ only differed in ski jumpers, compared to controls (patellar and Achilles tendon; +11% and +27%, respectively) and to water polo players (Achilles tendon only; +23%). Tendon size appears as an adjusting variable to changes in loading volume and/or intensity, possibly to preserve ultimate strength or fatigue resistance. However, uncoupled morphological and mechanical properties indicate that functional requirements may also influence tendon adaptations.

## Introduction

The prime function of tendons is to transmit forces from muscles to bones. Hence, tendons need to be strong enough to sustain high magnitudes of loading while their mechanical properties must remain functionally adequate for optimal muscle shortening or elastic energy storage. These diverse requirements are met across mammalian species with a broad range of tendon designs and properties, and seem maintained via a remarkable capacity of adaptation to altered loading conditions.

Short-term *in vivo* studies nearly systematically report a tendon stiffening after resistance training [1–3] and in some cases, increases in cross-sectional area (CSA) indicated that tendon hypertrophy can also occur with mechanical loading [2, 4–6]. Comparison studies actually suggest that an increase in CSA is a consistent adaptive feature and may well be the ultimate one for tendons [7]. Yet the loading stimuli driving these adaptations are poorly understood. Current hypotheses based on structural integrity or functional requirements originate from cross-species observations on tendon design (see [8] for review).

As structural elements of the musculoskeletal system, tendons need to fulfill design requirements based on the ratio of their peak operating stress to their ultimate stress (i.e. safety factor) [9–11]. However, safety factor varies between tendons, depending on their function and daily stresses. Most tendons are in fact thicker than required for a suitable safety factor [12]. Other tendons, those required to store and release larger amount of elastic energy are slenderer and operate at larger stress levels, with low safety factors. On the basis of these observations, Ker and colleagues [12] connected tendon design to stiffness requirements and the necessity for the muscle to operate at an optimal length. Lastly, an additional factor may independently affect tendon design. Fatigue damage incurred during cyclic loading seems relatively high in tendons [13–15] and is related to daily stress levels [16]. As a result, tendon resistance to fatigue damage needs to match loading conditions and may also play a role in the determination of tendon morphological and mechanical properties.

The collective suitability of these theories to describe tendon adaptations to training has never been examined. Current knowledge on these adaptations is rather fragmented into studies yielding partial information on certain tendon properties or on the effects of certain types of loading (e.g. [17–22]). The present study proposes to test for the first time mechanical and morphological properties of both patellar (PT) and the Achilles tendons (AT) in various athletes. The main purpose was to investigate whether specific loading patterns associated with different sports would be reflected by tendon properties. A secondary aim was to examine whether these differences could be interpreted in relation with paradigms of structural integrity or functional requirements.

Literature reviews [7, 23] indicate that short-term (< 6 month) studies are insufficient to induce the marked adaptations seen after years of specific loading conditions [4, 17, 20, 21, 24]. Our study was therefore based on a cross-sectional comparison, from which we set out to identify the influence of function (effective load transmission vs. spring-like function) and stress levels (training intensity, volume) upon the tendon properties of elite athletes from different sports. Since neither functional nor structural requirements are independently imposed in physical activities, we identified three sports with contrasting tendon function and loading: Ski jumping, distance running and water polo. Lower limbs muscles of ski jumpers are required to produce high amounts of explosive contractions. We expected these athletes to display a high tendon stiffness, to account for their high operating stress and to favor power output with rapid energy release. Distance runners were selected because of the high volume of cyclic loading imposed to their patellar and Achilles tendons. Based on previous cross-sectional [4, 17, 25] and longitudinal [26] studies, tendon stiffness was expected to be lower in runners than in ski jumpers but higher than in sedentary controls, owing to their higher operating stress. Water polo players were included as the third group of athletes because of comparatively lower loading requirements of their lower limb tendons. The water medium limits energy storage and release and their tendons are mainly expected to transmit force effectively. However, lower limb tendons presumably experience lower stresses during swimming than land-based activities. For this reason we predicted a lower Achilles and patellar tendon stiffness in these athletes.

Hence, tendon CSA would supposedly be larger—compared to controls—in ski jumpers and runners and smaller in water polo players, because of differences in daily stress levels.

In summary, we hypothesized higher stiffness and CSA values in athletes whose tendons play an important role in power amplification or are subjected to high impact forces (i.e. runners and ski jumpers). In consideration of their loading pattern and volume, we also expected opposite observations in water polo players. Finally, distinct properties would be observed in high stress—Achilles—and low stress—patellar—tendons.

## Methods

### Subjects

Forty healthy adult male subjects were recruited to participate in this study: 10 world class ski jumpers, 10 highly trained endurance runners, 10 national level elite water polo players (Austrian Champions) and 10 sedentary individuals. The ski jumpers were members of the Austrian and German national teams and had been following their specific training regime for 10-to-18 years (13.9 ± 2.2 yrs on average). The pre-competition training phases of this group consisted of 8–10 sessions per week, with a focus on resistance training of the lower legs. Training content was optimized to favor high-intensity contractions, with low numbers of repetitions at high levels of resistance and explosive jumps. The training volume of endurance runners ranged from 60 to 100 km/week, which corresponds to approximately 31.400 loading cycles [27]. All runners had been training for the last 5-to-13 years (7.8 ± 2.6 yrs on average). Water polo player had practiced their sport in all-deep water pools 4-to-6 times per week, for 4-to-15 years (9.0 ± 3.5 yrs on average). Neither the runners nor the water polo players had participated in regular lower body resistance training. The control subjects had no prior history of “high load” activities such as resistance training, ball or racquet sports, bike or regular (>1/week) running. Control subjects were recruited within the University and the local community of the city of Salzburg, Austria. Exclusion criteria were self-reports of knee or ankle injuries that impeded maximal muscle contraction, any history of PT or AT pain, Osgood-Schlatter disease, cardiovascular disorders, medical history of diabetes, respiratory and neuromuscular diseases or disclosure of anabolic drug abuse. Subjects were textually and/or verbally contacted, informed about the experimental procedures, purposes, risks and benefits and voluntarily signed a written informed consent prior to participation. The research protocol conformed to the Declaration of Helsinki and was approved by the Ethics Committee of the University of Salzburg.

### Experimental design

A four-group, cross-sectional study design was conducted to determine the effect of long-term physical activity on tendon properties. All subjects were asked to refrain from any vigorous activity (e.g. resistance training, running, jumping) for 24 hours leading up to the testing session, but to maintain their normal diet. Tests were performed on either leg, according to a prior block randomization based on leg dominance. Additionally, the sequence of tests at the knee and ankle joints was randomly chosen. Ski jumpers and runners were tested out of competition period. Water polo players were tested at the end phase of their season but at least three days following the last competition event. Interday reliability was assessed from the data of the control group, tested twice between three-to-five days at similar time of day (± 2 h). Each measurements and analyses were done by the same investigators.

### Maximal voluntary contraction

Testing sessions to measure maximal voluntary contractions (MVC) were preceded by a 10-min supervised warm-up protocol consisting of cycling on a stationary ergometer (Heinz Kettler GmbH and Co. KG, Ense-Parsit, Germany) at a sub-maximal intensity of 1.5 W/kg and a pedal rate of 70 rpm. This moderate intensity was well suited for both athletes and untrained without fatiguing the participants. Subjects were then seated and fastened on a rigid isokinetic dynamometer chair (IsoMed 2000 D&R Ferstl GmbH, Hemau, Germany). Knee muscle torque was tested at a joint angle of 90° (0° corresponding to full extension). Device calibration, positioning and fastening of the subjects on the dynamometer for estimating knee extensor and flexor muscles torque have previously been reported in detail [28]. Isometric plantar- and dorsiflexions were performed in prone position with the hip and knee fully extended and the ankle joint at 90° (anatomical position). The dynamometer axis was carefully aligned with the rotation axis of the ankle joint. Subject sliding and ankle joint rotation were minimized by adjusting shoulder pads, hip and footplate straps.

Subjects were familiarized with each of the testing procedures with practice trials and detailed instructions. Two maximal isometric contractions were performed with the knee or ankle extensors/flexors as appropriate. Resting periods lasted for one minute between attempts of the same test and two minutes between tests. To determine PT and AT properties (see *patellar and Achilles tendon mechanical properties*), isometric ramp contractions to maximal exertion were recorded. Tendon force was calculated offline as the sum of agonist and antagonist muscles’ torque and divided by the estimated PT or AT moment arm length as appropriate. Antagonist muscle torque was estimated from surface electromyography (sEMG) recordings [29]. Surface electrodes (Ag/AgCL; 120 dB, Input impedance: 1200 GOhm; 10 mm diameter, 22 mm spacing, Biovision, Wehrheim, Germany) were apposed on the biceps femoris and tibialis anterior muscles’ belly. Raw sEMG signal was filtered offline using a second-order Butterworth filter, by using a cutoff frequency of 10 and 300 Hz. Maximal, agonist, sEMG activity was quantified with root mean square calculations over a 0.5-s period around peak torque during knee flexion and dorsiflexion. Consequently, antagonist flexion torque was estimated by assuming linearity between sEMG and isometric torque production. Patellar tendon moment arm was estimated as a function of upper segment length [30]. The lever arm length of the AT was defined as the shortest distance between the joint axis of rotation and the tendon line of action. To obtain this parameter the distance between the outermost tip of the medial malleolus and the posterior surface of the calcaneus was measured externally with a ruler. Additionally, the calcaneal insertion of the tendon was located and imaged with ultrasound scanning. The moment arm length was calculated subsequently, by subtracting the depth of the tendon midline from the malleolus-calcaneus distance. Trials with the highest torque were normalized to body mass^2/3^ and used for further analysis. All tests were performed with strong verbal encouragement and visual feedback.

### Patellar and Achilles tendon morphology

**Fig 1:**
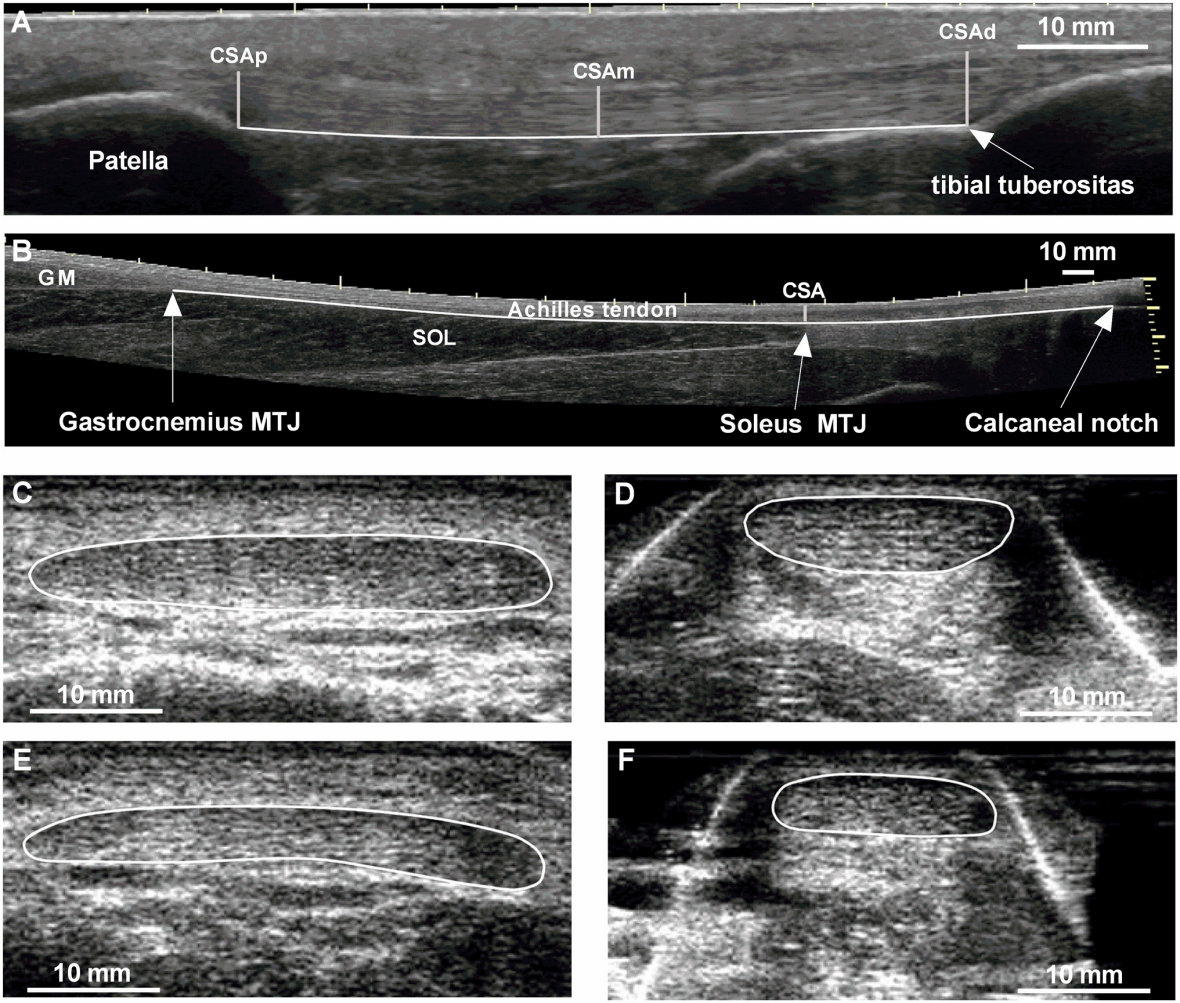
Ultrasound images of patellar and Achilles tendons length (A, B) and cross-sectional area (C, D). Sagittal plane views show the identifying landmarks used in measurement of Achilles tendon length (A) and patellar tendon length (B). Transversal ultrasound scans show outlined cross-sectional area of the patellar tendon mid-region (C, E) and of the Achilles tendon (D, F) in one distance runner (C, D) and one swimmer (E, F). CSAp, cross-sectional area proximal; CSAm, cross-sectional area medial; CSAd, cross-sectional area distal; MTJ, myotendinous junction; GM, gastrocnemius medialis; SOL, Soleus.

Patellar tendon length was measured via panoramic ultrasound scans (12L-SC, 8.0- to 13.0 MHz transducer, LOGIQ *e* Ultrasound—BT12, General Electric Company) at knee joint angle of 90° (0° corresponding to full extension), as the distance between tendon insertions on the patella apex and the tibial tuberosity. The AT length was conducted on subjects lying prone with their legs fully extended. Longitudinal panoramic scans of the AT spanned from the distal aspect of the calcaneal notch to the gastrocnemius myotendinous junction (Fig 1). Tendon cross-sectional area was measured from transversal ultrasound scans (linear array transducer 5 cm, LA523, 10- to 15-MHz transducer, MyLab25, Esaote, Genoa, Italy). The reliability of this technique has been shown previously [31]. Patellar tendon CSA was obtained as the average of two measurements performed at the proximal (CSAp) and distal (CSAd) insertions and at tendon mid-length (CSAm). The mean CSA of these three scan positions was used for further analysis. Because of technical limitations related to transversal scans of the Achilles tendon, cross-sectional area was only measured at one site in this tendon, at the distal end of the soleus muscle, where a high reliability can be obtained [32]. All measurements were performed offline with an image-processing program (ImageJ, Rasband, W.S., National Institutes of Health, Bethesda, MD, USA). Patellar tendon length was measured as the distance between the proximal insertion of the patellar tendon and the tibial insertion. The length of the AT was defined as the tendinous part running between the calcaneal insertion and the gastrocnemius myotendinous junction (Fig 1). To account for inter-individual differences in physical characteristics of the subjects, tendon CSA was normalized to body mass^2/3^ (assuming isometric scaling between tendon and body mass; nCSA) and tendon length was normalized to leg length. This approach was necessary to account for the close relation between body weight and tendon CSA [17] and by the significant differences in physical characteristics between groups (Table 1). The experimenter (HPW) performing all ultrasonographic measurements maintained a minimal probe pressure to avoid local deformation of the skin and underlying structures of interest. The intraclass correlation coefficients (ICC) of tendon length measurements were 0.90 and 0.98, with typical errors of 0.58 mm and 0.09 mm, respectively, for the AT and PT. The ICCs and typical errors for the PT CSA measurements were 0.91 and 2.0 mm^2^, respectively, and 0.93 and 2.4 mm^2^, respectively, for the AT CSA.

**Table 1:** Anthropometric characteristics of subjects.

	Ski Jumper	Runner	Water Polo	Control	F_(3;35)_	P-Value	*η*^*2*^*-Value*
Sample size	10	10	9	10			
Age (yrs)	22.2 ± 2.9^†††^***	31.5 ± 4.6	24.2 ± 3.2^†††^***	31.0 ± 5.1	13.08	<0.001	0.53
Body height (cm)	176.3 ± 4.5	180.9 ± 8.2	182.4 ± 6.5	182.9 ± 7.2	1.96	0.140	0.14
Body mass (kg)	64.3 ± 3.9^†^^§§§^***	72.8 ± 7.6^§^	84.3 ± 10.8	83.9 ± 12.3	10.72	<0.001	0.48
BMI (kg/m^2^)	20.7 ± 1.0^§§§^***	22.2 ± 1.7^§^*	25.3 ± 2.8	25.0 ± 2.8	10.33	<0.001	0.47

Values are expressed as mean ± SD. BMI, body mass index;

^†^ P < 0.05;

^†††^ P < 0.001 compared with runners;

^§^ P < 0.05;

^§§§^ P < 0.001 compared with water polo players;

* P < 0.05;

*** P < 0.001 compared with controls.

### Patellar and Achilles tendon mechanical properties

The details of the method used to assess PT properties via isometric ramp contraction, including its reliability, have been reported previously [33]. In brief, PT stiffness and Young’s modulus were estimated from isometric contractions at a constant loading rate (110 Nm/s) [33]. Each subject was familiarized with the task of following the displayed loading rate as accurately as possible until reaching their maximal voluntary contraction level. Two ultrasound recordings per testing condition were retained for analysis. Elongation of the PT was measured by tracking the displacement of the patella apex and the tibial antero-superior aspect with a software for semi-automatic video analysis (Tracker 4.87, physlets.org/tracker/).

Similarly, the displacement of the gastrocnemius AT insertions during isometric ramp contractions was measured to obtain its elongation. Proximally, the probe was placed on the gastrocnemius medialis MTJ approximately 2 cm medial from the border between medial and lateral gastrocnemius. The muscle-tendon junction displacement was tracked offline in each video frame. Artefactual movements of the probe or the heel were recorded with a single high-speed video camera (JVC GC-PX100BEU at 200 Hz) perpendicular to the motion and corrected offline with 2D motion analysis. To this end, reflective markers were attached to the ultrasound probe handle (MyLab 25, see above), the calcaneous and the dynamometer footplate.

Offline data synchronization and processing were performed using custom-written Matlab code (version R2013a; The MathWorks Inc., Natick, Massachusetts, USA). Surface EMG and dynamometer signals were simultaneously sampled at a sampling frequency of 2 kHz (Biovision, Wehrheim, Germany). Ultrasonographic and kinematics sequences were synchronized from an electrical pulse and a flash light simultaneously sent during data acquisition to the ultrasound system and the video camera, respectively. Torque signals were offset and smoothed using a digital second-order, zero-lag Butterworth filter with a cutoff frequency of 15 Hz.

Load-deformation data obtained for the PT and AT with this procedure were then fitted with a second order polynomial (all relations retained for analysis had a coefficient of variation R^2^ higher than 0.95). Ramp contractions with a produced torque lower than 80% of MVC were discarded to ensure reaching the elastic region of the tendon load-deformation curve. Tendon stiffness was calculated as the slope of this curve at force levels corresponding to i) the individual last 10% of the maximal force of the ramp contractions and ii) the maximal common force level of all subjects. The force range common to all subjects was 3600 N to 4000 N for the PT and 2250 N to 2500 N for the AT, respectively. Similarly to tendon CSA normalization and to isolate the influence of different training types, stiffness was normalized to body mass^2/3^. Young’s modulus was calculated as the slope of the stress-strain relationship. Stress was calculated by normalizing tensile tendons’ force by mean PT or AT CSA, as appropriate. Strain was obtained as the elongation of PT and AT relative to their resting length. The interday reliability of tendon stiffness yielded intraclass correlation coefficients of 0.86 and 0.83, with typical errors of 185 N·mm^-1^ and 20 N·mm^-1^, respectively, for the PT and AT.

### Statistics

Data were checked statistically for normality of distribution using the Kolmogorov—Smirnov test. Between-group differences in anthropometric parameters, MVC and PT or AT properties were tested with a one-way analyses of variance (ANOVA). In case of significant between-group effect, a Tukey corrected post hoc tests was performed to assess statistical significance of differences between mean values of the four groups. Effect size was defined according to Cohen [34], with the standardized effect (η^2^) being small for η^2^ > 0.1, medium for η^2^ > 0.25, and large for η^2^ > 0.4. All analyses were conducted using SPSS V.22.0 (SPSS Inc., Chicago, Illinois, USA) and the critical level of statistical significance was set at an α level of 0.05.

## Results

### Anthropometric characteristics

Data from one water polo player were discarded because of the late disclosure of prior inclusion of resistance training in his training routine. Physical characteristics of the remaining 39 subjects are presented in Table 1. Ski jumpers and water polo players were younger than runners and controls. Body height was generally similar between groups but ski jumpers were lighter than other subjects. Body mass index was also different, being lower in ski jumpers and runners than in water polo players and controls. For simplicity, the term “normalized” is hereinafter used in combination with “force”, “tendon cross-sectional area” or “stiffness” instead of “normalized to body mass^2/3^.

### Muscle force

The maximal isometric force produced during knee extension did not differ between groups (F_(3;35)_ = 0.50, *P* = 0.682, η^2^ = 0.04). However, when normalized to body mass^2/3^, knee extension force (main effect: F_(3;35)_ = 3.84, P = 0.018, η^2^ = 0.25) was higher in the ski jumpers compared to controls (+23%, Q_(4;35)_ = 3.29, P < 0.012, η^2^ = 0.33), whereas no significant difference was observed between other groups.

**Fig 2:**
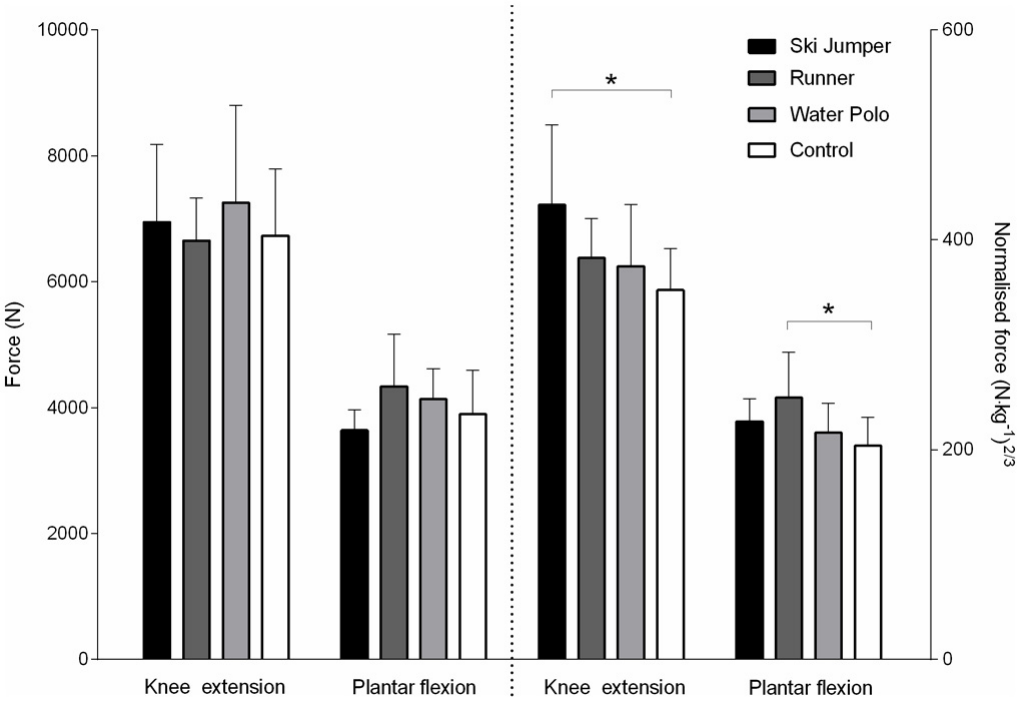
Raw and normalized (body mass^2/3^) maximal force values recorded during knee extension and plantarflexion. Values are mean ± SD. * P < 0.05.

Similarly, raw values of maximal isometric force (F_(3;35)_ = 2.40, P = 0.085, η^2^ = 0.17) produced during plantarflexion did not differ significantly between groups but normalized force was 22% (Q_(4;35)_ = 3.28, *P* = 0.012, *η*^*2*^ = 0,31) higher in runners than in the controls (main effect: F_(3;35)_ = 3.85, P = 0.018, η^2^ = 0.25). There were no other significant differences between groups (Fig 2).

### Tendon morphological properties

**Fig 3:**
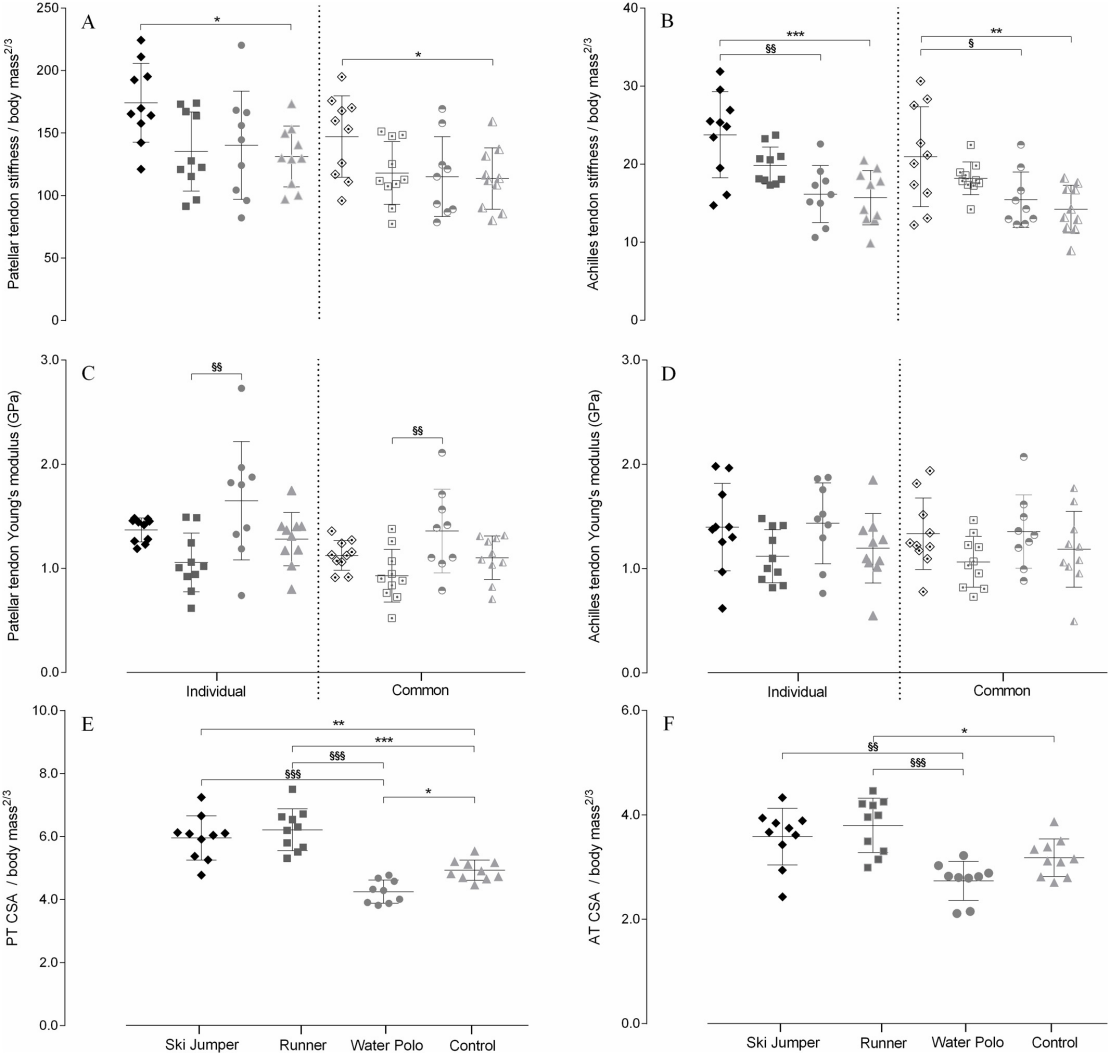
Between-group comparisons of morphological and mechanical properties for the patellar (A, C, E) and Achilles (B, D, F) tendons. Normalized stiffness (A, B), Young’s modulus (C, D) and normalized cross-sectional area (E, F) are presented. Scatter plots with mean ± SD bars for ski jumpers *(rhombus)*, runners *(square)*, water polo players *(circle)* and controls *(triangle)* are shown. *Filled symbols* indicate tendon properties evaluated at individual maximal force level and *semi open symbols* indicate tendon properties evaluated at maximal force level common to all subjects (3.600–4.000 N for PT and 2.250–2.500 N for AT). ^§^ P < 0.05, ^§§^ P < 0.01, ^§§§^ P < 0.001 compared with water polo players.* P < 0.05, ** P < 0.01, *** P < 0.001 compared with controls.

Neither raw nor normalized PT length differed between groups (Table 2). Raw measurements of PT CSA were significantly higher in runners compared to water polo players (+30%, Q_(4;35)_ = 5,08, *P* < 0.001, η^2^ = 0.58) and controls (+15%, Q_(4;35)_ = 2.90, *P* = 0.031, η^2^ = 0.32), while no difference was found for other group comparisons. However, when taking body mass into account, other numerical differences in PT CSA became significant. Hence, normalized CSA of ski jumpers and runners was larger than that of water polo players (+33%, Q_(4;35)_ = 6.80, *P* < 0.001, η^2^ = 0.72; +39%, Q_(4;35)_ = 7.82, *P* < 0.001, η^2^ = 0.78, respectively) and controls (+21%, Q_(4;35)_ = 4.20, *P* = 0.001, η^2^ = 0,49; +26%, Q_(4;35)_ = 5.25, *P* < 0.001, η^2^ = 0.62, respectively), but not different between ski jumpers and runners. Additionally, normalized tendon CSA of water polo players was smaller than that of controls (-9%, Q_(4;35)_ = 2.71, *P* = 0.048, η^2^ = 0.52; main effect: (F_(3;35)_ = 26.54, *P* < 0,001, η^2^ = 0.70) (Fig 3).

**Table 2:** Knee extension maximal torque and patellar tendon properties.

	Ski Jumper	Runner	Water Polo	Control	F_(3;35)_	P-Value	*η*^*2*^*-Value*
Isometric torque (N·m)	233 ± 45	223 ± 27	239 ± 54	225 ± 40	0.23	0.878	0.02
Stiffness (N/mm)	2792 ± 477	2348 ± 548	2704 ± 872	2509 ± 511	1.04	0.388	0.08
Young's modulus (GPa)	1.4 ± 0.1	1.1 ± 0.3^§^	1.7 ± 0.7	1.3 ± 0.3	4.35	0.011	0.27
Strain (%)	8.4 ± 1.7	8.2 ± 2.3	8.9 ± 1.6	8.6 ± 1.4	0.32	0.814	0.03
Stress (MPa)	76.9 ± 7.6^†^	62.4 ± 6.3^§§§^	87.6 ± 17.1^†^*	71.6 ± 9.1	9.24	<0.001	0.44
PT length (cm)	4.6 ± 0.6	4.8 ± 0.4	5.0 ± 0.5	4.8 ± 0.6	1.17	0.334	0.09
nPT length (a.u.)	1.0 ± 0.2	1.1 ± 0.1	1.2 ± 0.2	1.1 ± 0.1	1.63	0.200	0.12
PT mean CSA (cm^2^)	1.0 ± 0.1	1.1 ± 0.1^§§§^*	0.8 ± 0.1	0.9 ± 0.1	8.67	<0.001	0.43

Values are mean ± SD. Stiffness, Young's modulus and strain are measured at the highest individual force level. Stress was measured from the highest MVC trial. nPT length, normalized patellar tendon length; CSA, cross-sectional area; F, P and η^2^ show the interaction effect between group;

^†^ P < 0.05 compared with runners;

^§^ P < 0.05;

^§§§^ P < 0.001 compared with water polo players;

* P < 0.05 compared with controls.

Achilles tendon length was similar across groups, with the exception of the smaller tendon length found in ski jumpers (-13%, Q_(4;35)_ = 2.93, *P* = 0.029, η^2^ = 0.41) when compared to water polo players. However, no difference in this variable was found when normalized to leg length (Table 3). Raw measurements of AT CSA indicated that this tendon was thinner in water polo players than in runners (-27%, Q_(4;35)_ = 3,81, P = 0.003, η^2^ = 0.48), with no other difference between groups. After normalization to body mass, AT CSA was smaller (main effect: F_(3;35)_ = 9.91, P < 0.001, η^2^ = 0.46) in water polo players than in all other athletes groups (SJG: -23%, Q_(4;35)_ = 4.02, *P* = 0.002, η^2^ = 0.48; RG: -28%, Q_(4;35)_ = 5.05, *P* < 0.001, η^2^ = 0.60). Normalized AT CSA was also significantly larger in runners (+20%, Q_(4;35)_ = 3,03, *P* = 0.028, η^2^ = 0.26) than in the controls. Other elite athletes did not differ from the control group and there was no difference between the ski jumpers and the runners.

**Table 3:** Plantarflexion maximal torque and Achilles tendon properties.

	Ski Jumper	Runner	Water Polo	Control	F_(3;35)_	P-Value	*η*^*2*^*-Value*
Isometric torque (N·m)	188 ± 15	226 ± 46	228 ± 28	217 ± 38	2.95	0.046	0.20
Stiffness (N/mm)	380 ± 85	345 ± 47	309 ± 68	299 ± 66	2.89	0.049	0.20
Young's modulus (GPa)	1.4 ± 0.4	1.1 ± 0.3	1.4 ± 0.4	1.2 ± 0.3	1.83	0.160	0.14
Strain (%)	6.5 ± 1.9	6.1 ± 1.3	6.1 ± 1.5	6.2 ± 1.5	0.12	0.945	0.01
Stress (MPa)	71.2 ± 10.8	66.4 ± 13.4	81.9 ± 13.0	66.1 ± 14.4	3.02	0.043	0.21
AT length (cm)	20.7 ± 1.5^§^	23.2 ± 3.1	23.8 ± 2.5	23.1 ± 2.0	3.45	0.027	0.23
nAT length (a.u.)	4.4 ± 0.2	4.8 ± 0.5	4.8 ± 0.4	4.7 ± 0.4	1.54	0.222	0.12
AT CSA (cm^2^)	0.6 ± 0.1	0.7 ± 0.1^§§^	0.5 ± 0.1	0.6 ± 0.1	5.15	0.005	0.31

Values are mean ± SD. Stiffness, Young's modulus and strain are measured at the highest individual force level. Stress was measured from the highest MVC trial. nAT length, normalized Achilles tendon length; CSA, cross-sectional area; F, P and η^2^ show the interaction effect between group;

^§^ P < 0.05;

^§§^ P < 0.01 compared with water polo players.

### Tendon mechanical and material properties

Tendon mechanical and material properties calculated at maximal common or individual force levels yielded similar comparative differences. Results obtained with both approaches are reported but, for the sake of simplicity and a better functional relevance, only values calculated at individual maximal forces were retained in tables and figures and for the discussion.

Raw values of PT stiffness did not differ between groups. However, after normalization to body mass, the PT stiffness of ski jumpers was found higher than in controls (individual: +33%, Q_(4;35)_ = 3.01, P = 0.023, η^2^ = 0,39, main effect: F_(3;35)_ = 3.81, *P* = 0.018, η^2^ = 0.25, common force level: +19%, Q_(4;35)_ = 2.74, P = 0.047, η^2^ = 0.28, Fig 3), while no difference was observed between other groups. Apart from the lower PT Young’s modulus (individual: -40%, Q_(4;35)_ = 3.56, P = 0.002, η^2^ = 0.29; common: -35%, Q_(4;35)_ = 3.42, P = 0.005, η^2^ = 0.29) in runners than in water polo players, PT material property did not differ between groups. Similarly to PT stiffness, AT stiffness did not differ between groups, with the notable exception of ski jumpers having stiffer AT than controls when looking at normalized values (individual: +51%, Q_(4;35)_ = 4.58, *P* < 0.001, η^2^ = 0,46; common: +43%, Q_(4;35)_ = 3.02, *P* = 0.028, η^2^ = 0,25, main effect: F_(3;35)_ = 8,95, *P* < 0.001, η^2^ = 0.43, Fig 3). Additionally, normalized AT stiffness of ski jumpers was also higher than in water polo athletes (individual: +37%, Q_(4;35)_ = 4.21, P = 0.001, η^2^ = 0.42; common: +35%, Q_(4;35)_ = 2.83, P = 0.046, η^2^ = 0.24). There was no difference in AT Young’s modulus between elite athletes and controls, whether this variable was calculated at individual (F_(3;35)_ = 1.83, *P* = 0.160, η^2^ = 0.14) or common (F_(3;35)_ = 1.69, *P* = 0.186, η^2^ = 0.13) force level.

Tendon stress ranged 40-to-94 MPa and 33-to-110 MPa for the PT and AT, respectively. Tukey-corrected post-hoc analysis indicated significantly higher PT stress levels in ski jumpers (+23%, Q_(4;35)_ = 3.04, *P* = 0.022, η^2^ = 0.54) and water polo players (+40%, Q_(4;35)_ = 5.15, *P* < 0.001, η^2^ = 0.53) than in runners (Table 2), with no other difference between athlete groups. Patellar and Achilles tendons strain did not significantly differ between groups. Normalised PT and AT properties are summarized in Fig 2, with absolute values reported in Tables 2 and 3 for the PT and AT, respectively.

## Discussion

The main finding of the present study is that elite athletes involved in different sports display distinctive sets of mechanical, material and morphological properties of the patellar and Achilles tendons. When normalized to body mass, tendon CSA was larger in athletes with high intensity or volume of loading and was lower than controls in athletes with lower magnitude of loading or impact-like activities (water polo players). Yet differences in CSA were not mirrored by differences in tendon stiffness in every group. Taken together, these results suggest that theories formulated to explain tendon design may apply in the context of adaptation to altered mechanical loading: Structural integrity may be maintained via hypertrophy but tendon stiffness and CSA can be uncoupled, presumably to fulfill functional requirements. Contrary to our hypothesis, athletes from different groups displayed similar differences in patellar and Achilles tendon properties. These findings suggest that mechanical constraints inherent to daily activities may override the functional specificity of lower limb tendons. Some of the athletes were younger (~7–9 yrs on average, Table 1) than others. Although aging reportedly affects tendon properties [35–37], an age difference within the present range of 20-to-30 yrs is unlikely to have influenced our results. This assumption is supported by a previous study showing a lack of significant difference in Achilles tendon maximal strain between subjects aged 20–27 yrs and aged 30–38 yrs [37].

### Activity-dependent tendon cross-sectional area

The larger tendon nCSA measured in ski jumpers and runners is consistent with previous observations from individuals exposed to long term high volume [17, 24], or simply high intensity [20, 21], exercise. The smaller patellar tendon nCSA measured in water polo players in the present study—and the similar trend observed for the Achilles tendon—is important in this context. This relatively reduced CSA may be explained by a lower daily occurrence of high stresses. Water polo players spend a remarkable amount of their daily time in water. Their tendons are exposed to high number of loading cycles but impact and, possibly, the magnitude of muscle contractions are reduced in the water medium. In line with this, the present data show for the first time that in some athletes, tendon CSA can even be lower than in untrained individuals, despite years of athletic practice. These observations complete the relation between tendon daily loading and size suggested by previous work (see Wiesinger et al [7] for review) and the present results, supporting the hypothesis of tendon size as an adjusting variable to changes in loading.

This interpretation is congruous with the normalization of tendon CSA to body mass to reflect adaptations to external loading. Accordingly, the present differences in nCSA are compatible with the hypothesis of adaptive mechanisms drawn towards maintaining tendon structural integrity. The typical physical preparation of ski jumpers includes several types of jumping and hopping that involve considerable patellar [38–40] and Achilles tendon [41] forces. Tendon stresses imposed by these types of activity are close to theoretical fracture stresses established *in vitro* at about 100 MPa [12, 42, 43]. Similarly, running performed over 60 to 100 km/week by the runners’ group, repetitively stretches their Achilles tendon to ~6% of strain [44, 45] during ~31.400 loading cycles [27] per week, while the patellar tendon was only slightly less loaded [46]. We could not find estimations of tendon loading for water polo players or for individuals such as our controls but attributing a higher loading volume to runners and ski jumpers seems a fair assumption. Thus, the need to maintain a sufficient ultimate strength or to reduce operating stresses may drive tendon hypertrophy in athletes subjected to high magnitude (ski jumpers) or high volume (runners) of loading. Ultimate strength cannot be measured *in vivo*. Assuming that the relationship observed between ultimate stress and Young’s modulus [47] holds true, the similar Young’s modulus measured in ski jumpers and runners would suggest that tendon strength was not particularly higher in the former. Yet this interpretation should be made with caution, because of the difficulties to assess Young’s modulus accurately *in vivo* [48]. Determining this variable or ultimate stress with more accuracy is required to ascertain the influence of loading pattern on tendon strength. On the other hand, a reduction in operating stresses would be consistent with measurements of maximal stress, which were found lower or equal in both tendons of ski jumpers and runners, compared to water polo players and controls. Should ultimate stress be similar in all groups, these results would indicate that the safety factor—and structural integrity—is increased or maintained in individuals with higher intensity or volume of loading.

### Uncoupling between tendon cross-sectional area and stiffness

On the one hand, a higher safety factor would be consistent with the training regime of ski jumpers and runners. On the other, all athletes presented similar maximal isometric forces and most mammalian tendons seem to have higher safety factors than expected from muscle force [12]. The latter observation led Ker [12] and colleagues to argue that tendon thickness is set to ensure sufficient stiffness, rather than sufficient strength. However, contrary to logical predictions from the relation between cross-sectional area and deformation to uniaxial force, differences in CSA were not mirrored by differences in stiffness in the present study.

Raw values of PT and AT tendon stiffness were similar across groups despite differences in CSA. When adjusted to body mass, tendon stiffness was higher in ski jumpers than in controls but did not differ between runners and other groups, despite their larger tendon nCSA (Fig 3). Similarly, the lower nCSA found in water polo players than in controls (patellar tendon) or other athletes (both tendons) was not reflected by lower stiffness values, with or without normalisation to body mass. This dissociation between size and stiffness is consistent with previous observations [17] but is difficult to conciliate with the “theory of thick tendons” proposed to elucidate tendon design [12]. Furthermore, the counterpart of this theory, the slenderness of tendons acting as springs due to the necessity to be compliant enough, does not seem to fit with the larger tendon CSA of runners. Taken together, these findings suggest that tendon adaptations may not be explained by the necessity to increase ultimate strength or to regulate stiffness alone. Since a higher tendon loading volume seems imposed to runners than to ski jumpers, we speculate that tendon hypertrophy may occur in the former to counteract a high rate of fatigue damage. Because of the relation between stress level and tendon damage rate [14], an increase in tendon CSA routine fatigue damage. Hence, fatigue resistance to submaximal but repeated tendon stresses may be maintained via hypertrophy independently from functional requirements for tendon stiffness.

The dissociation between tendon stiffness and CSA is theoretically connected to differences material properties. However, this assumption was only partially consistent with our calculations of tendon Young’s modulus. For instance, the lack of difference between tendon tensile modulus of ski jumpers and that of other athletes fits with their comparable ratio of stiffness to CSA. Likewise, the higher patellar tendon Young’s modulus of water polo players, compared to runners, fits their lower CSA relative to stiffness. Yet a significant difference in Young’s modulus would have also been expected between ski jumpers and runners, to reflect their different stiffness, relative to tendon CSA. Additionally, despite similar numerical patterns found in both tendons, no between-group difference in modulus was significant for the Achilles tendon. The methods currently used to obtain tendon Young’s modulus *in vivo* are notably hindered by the summed limitations inherent to stiffness and CSA measurements. A larger sample size may have enabled overcoming these limitations and detecting expectable differences, which did not reach significance in the present results. Generally, the interpretation of the present results is based on the assumption that between-group differences reflect adaptive mechanisms. Such an assumption is partly supported by training studies (see Introduction section) but additional investigations are needed to substantiate our conclusions.

### Tendon specificity?

In agreement with earlier descriptions [49, 50], the patellar tendon was systematically found thicker and stiffer than the Achilles tendon. As in cross-species comparisons [12], these differences have been ascribed to the contrasting requirements for each tendon to effectively transmit force or to store and release elastic energy. Some authors have observed sport-specific and expertise-specific differences in tendon stiffness, with some reports even indicating that higher stiffness values may be beneficial to performance for a particular tendon, whereas the opposite would apply to another one [19, 22]. Accordingly, we expected group differences to be more pronounced in either tendons depending on the requirements of athletic activities. However, relative differences between groups were similar in both tendons, for all main outcome variables (e.g. Fig 3). The similar patterns of properties measured in patellar and Achilles tendons suggest that adaptations to daily loading can take place independently from tendon type and function.

## Limitations

The Achilles tendon consists of two morphologically distinct parts, with different longitudinal strain (e.g. [51]) and perhaps, different adaptive responses to increased loading. Our results concern the full AT and additional experiments are required to ascertain that similar patterns are found in the free section of the AT. Additionally, Achilles tendon CSA was only measured at one site, corresponding to the proximal end of the so-called “free” tendon. Several authors have shown that hypertrophy can occur to different extent along the length of tendons [5, 52]. The measurement location chosen in the present study (~ 4 to 8 cm above calcaneal insertion) seems to coincide with regions of the Achilles tendon displaying athlete-specific differences [18] but this limitation should be taken into account when interpreting our data.”

## Perspectives

The present findings clearly suggest that sports specific loading patterns applied to PT and AT bring about distinctive sets of tendon properties. Are these presumed adaptations related to structural integrity or functional requirements? Both factors may be involved. Stiffness measurements—and their dissociation from tendon CSA—are consistent with the theory of thick tendons [12], placing mechanical properties and perhaps the operating range of muscles at the centre of adaptive mechanisms. However, nCSA seeming commensurate with daily loading, a reduction in ultimate or daily stress can certainly be ascribed to training-induced changes in tendon size. Whichever adaptive mechanisms may be, the similar sets of tendon properties measured in PT and AT are interesting, for they suggest that adaptations to chronic loading may override tendon specificity. The cross-sectional design of this study was necessary to get a glimpse of long-term tendon adaptations, important stepping stone to our understanding of tendon physiology pathophysiology. Future studies are needed to confirm these interpretations with longitudinal designs and to complement these findings by examining properties of energy storage and release.

## Supporting Information

S1 Table Sport Specific Tendon Properties. (XLSX)
